# Personalizing solidarity? The role of self-tracking in health insurance pricing

**DOI:** 10.1080/03085147.2019.1570707

**Published:** 2019-03-19

**Authors:** Liz McFall

**Affiliations:** Liz McFall, Edinburgh Futures Institute & Sociology, University of Edinburgh, 21 George Square, Edinburgh, EH8 9LD, United Kingdom.

**Keywords:** Patient Protection and Affordable Care Act 2010 (ACA), Obamacare, Oscar Health, solidarity, risk, self-tracking

## Abstract

Can data-driven innovations, working across an internet of connected things, personalize health insurance prices? The emergence of self-tracking technologies and their adoption and promotion in health insurance products has been characterized as a threat to solidaristic models of healthcare provision. If individual behaviour rather than group membership were to become the basis of risk assessment, the social, economic and political consequences would be far-reaching. It would disrupt the distributive, solidaristic character that is expressed within all health insurance schemes, even in those nominally designated as private or commercial. Personalized risk pricing is at odds with the infrastructures that presently define, regulate and deliver health insurance. Self-tracking can be readily imagined as an element in an ongoing bio-political redistribution of the burden of responsibility from the state to citizens but it is not clear that such a scenario could be delivered within existing individual private health insurance operational and regulatory infrastructures. In what can be gleaned from publicly available sources discussing pricing experience in the individual markets established by the Patient Protection and Affordable Care Act 2010 (ACA), widely known as ‘Obamacare’, it appears unlikely that it can provide the means to personalize price. Using the case of Oscar Health, a technology driven start-up trading in the ACA marketplaces, I explore the concepts, politics and infrastructures at work in health insurance markets.

Today the average American’s health insurance payments fluctuate once a year. Imagine if that rate changed each *day*, determined in part by a sensor-rich gadget on the wrist. (Olson, 2014)

Data mining and monitoring not only allow insurers to price policies more accurately, but also enable them to modify customers’ behaviour. (*The Economist*, 2015)

## Introduction

How attentive to the personal should price be? Personalized products and services, the bespoke, tailored, monogrammed and unique are usually prized but prices are different. Prices should be general, they are tied to the qualities and quantities of a product consumed, but within certain tolerated variations they should be, or at least appear to be, the same for all buyers. This principle has a relatively short history and there have always been market spaces in which price is too fluid, too complex or opaque to expose patterns in its variations. Insurance is a case in point. Where the product priced is risk, and the product sold is security, there are easy arguments that price must vary. In private health insurance it is accepted that individual prices will vary according to age, behaviour and health indicators in the interests of statistical or ‘actuarial’ fairness (Arrow, 1963; Baker, 2010; Leaver, 2015; Meyers & Van Hoyweghen, 2018). This idea of actuarial fairness helps make the politics, the distributional stakes and consequences of insurance seem just technical. It is technical, but it is simultaneously social and political. The dynamics of personalized, behavioural, data-driven pricing expose this and subject insurance practices to renewed public, political and regulatory scrutiny.

New ‘big’ data forms – data that can be intimate and super-massive, dynamic and historical, scattered and integrated – present a global, grand challenge that is widely apprehended but less well understood empirically. Research on how this challenge is being handled in insurance is scant, notwithstanding the frequency with which insurance is invoked as an exemplar of the privacy, surveillance and societal concerns raised by big data. There are extant applications of behavioural tracking data, for example in car insurance, being used for risk assessment (McFall & Moor, 2018; Meyers, 2018) but the industry remains some way from the scenarios presented in the epigraphs. There are many sector and location specific reasons for this. In this paper I explain how the conceptual, regulatory and infrastructural practices of insurance act as barriers to risk personalization.

Insurance, whether nominally private or public, has a collective, social character. It can be thought of as a practical, organizational form of solidarity that allows payment responsibility for the risks of illness, injury, death, accident, crime, etc. to be shared. While prices or contributions charged to individuals vary, they vary by group, not individual, characteristics. As Swedloff puts it ‘insurers set prices by predicting the probability that any group of observationally identical individuals will suffer a loss … [they] individuate those prices by determining whether the particular observable characteristics of a particular insured correlate with particular harms’ (2015, p. 342). Big data working across an internet of connected things suggests the possibility of something different – a means of pricing based, not on observed, group characteristics, but on apps and devices tracking individual behaviour. This possibility is at odds with the infrastructures, that is, the conceptual and classificatory logics, the regulatory environment and the working practices that ‘sort out’ health insurance (Bowker & Star, 1999).

I concentrate specifically on the politically volatile individual insurance markets established by the Patient Protection and Affordable Care Act 2010 (ACA), popularly known as ‘Obamacare’, in the United States. In these market situations it is unlikely that self-tracking data can provide a meaningful proxy for individual risk. Insurers have an interest in self-tracking data and technologies that is connected to pricing but there is a proviso. Insurance pricing is not an exact science. Rather it is a delicate algebra connecting actuarial (mathematical) and non-actuarial (accounting) – factors. Price has to entice individuals away from bearing their own risks, comply with elaborate regulatory frameworks, balance premium income with investment income and reconcile a host of other costs and contingencies. This challenge has shaped the industry’s historically bearish attitude to reputation and branding in an attempt to stave off fluctuating consumer appeal and unwanted regulatory attention (McFall, 2011, 2014).

Still, close attention should be paid to how the industry deals with new technologies. ‘Big data’ technologies, correlative analyses and mythologies (boyd & Crawford, 2012) are industry ‘disruptors’. Who gets access to what data, what sorts of analyses are being conducted, to what ends? Already a handful of giant corporations with the means to analyze big data are benefitting while individuals, groups and governments, struggle to understand and regulate the consequences. Self-tracking data amplifies these concerns (Lupton, 2016a, 2016b; Neff & Nafus, 2016; O’Neil, 2016; Schüll, 2016) since, if insurance providers use it to personalize price they simultaneously personalize access to care. Given the numerous governmental efforts to embrace the emerging paradigms of digital, personalized healthcare as solutions to the global, triple challenge of managing costs, quality and access, it is important to scrutinize what payers want with self-tracking data. In what follows, I explore these issues beginning with a discussion of the conceptual foundations of solidarity as a solution to the social problem of insuring healthcare. I then use a documentary analysis of ACA policy and provisions to describe how the individual markets were set-up and how they incentivize personalization. I close with the case of Oscar Health, a company trading in the ACA marketplaces that has been closely associated with risk personalization.

## Concepts and organized practices of solidarity

It is the technical system’s coordinated ability to react, circumscribe a range of possible responses, to be the cause of its own outcomes that supports life - not the data. (Poon, 2016, p. 1090)

Martha Poon’s precise sentence goes straight to the infrastructural point. Data, no matter how super-massive, does nothing unless and until it is embedded in an infrastructure that can react to it in a coordinated way to achieve its own outcomes, its own solutions to identified problems. This is the core of the question of whether big data can personalize solidarity. The concept of solidarity can be traced back to the Roman law of obligations wherein it was defined as the unlimited liability of each individual member within a family or community to honour common debts (Bayertz, 1999; Prainsack & Buyx, 2017). By the early nineteenth century, solidarity functioned more as a political ideal, an ideal that became closely associated with the infrastructures and techniques of social or welfare provision, notably through insurance mechanisms. As the temper of post-war European social policy began to shift away from this ideal in the 1980s, a group of Foucauldian theorists re-examined this history, locating solidarity and the insurance mechanism within broader governmental rationalities or ‘governmentalities’ (Burchell *et al*., 1991).

In Jacques Donzelot (1988, 1991) recounting, welfare states emerged as the solution to the problem of how to define the role of the state after the 1848 European revolutions. The problem centred on the conflict between those who expected states to intervene to protect workers’ rights and those who expected minimal intervention. Solving this involved two operations that would constitute the welfare state. The first established a distinction between sovereignty and solidarity. The second saw the language of statistics transcend the language of rights, a process also carefully observed in the intellectual histories supplied by Ian Hacking (1990) and Theodore Porter (1996), among others. These moves helped transform solidarity from an abstract ideal to something that could be accomplished as a practice using the intellectual techniques and infrastructures of insurance. Solidarity, *inter alia,* becomes a means of defining the context, limits and justification of state intervention and the conceptual grounds for social legislation designed to absorb the greater risks faced by certain members of society. Insurance then is the ‘technique’ – a term I use to signal both the technologies and working practices involved – that realises this vision of solidarity. It also provides the first practical test of statistical knowledge as a tool for organizing the social (McFall, 2011; Poovey, 2002; Porter, 1996). In Ewald’s (1991) analysis, insurance allows the task of determining cause, blame or fault to be set aside. Instead the burden can be distributed across a community of members whose contributions can be fixed in explicit rules. The social problem can thereby be addressed by organizing interdependence rather than establishing responsibility for individual failings, faults and duties.

This vision of insurance as a welfare state technique is not, of course, the form US health insurance takes. Insurance has a formal generality that allows it to be versioned to suit many purposes. In O’Malley’s (1996) reckoning, this capacity to vary with governmental rationalities has assumed two main forms - socialized actuarialism, more or less conforming to welfare state ideals, and privatized actuarialism, more or less resembling the US model. In practice, political expediencies, contingencies and compromises mean that the working infrastructures of insurance combine socialized and privatized elements in most governmental contexts. These elements have become harder to disentangle amidst the welfare state reforms – designated loosely by terms such as marketization, privatization or neoliberalization – of the last 40 years. Material and substantial changes to the distribution of responsibility and risk continue. The interesting question is whether this also eradicates the solidaristic character of insurance.

Solidarity has come to connote redistributive forms of welfare policy (Baldwin, 1990; Lehtonen & Liukko, 2012; Prainsack & Buyx, 2017) that are clearly not expressed in all forms of insurance. Yet insurance, as defined by the infrastructures and practices through which it is enacted, always involves a solidaristic sharing or ‘pooling’ of risk. This is true even of US health insurance, a political context in which redistributive reforms are mobilized repeatedly as assaults on individual freedom. Attempts to establish universal coverage were politically fractious and bitterly fought, sometimes on surprising lines, throughout the twentieth century (Dobbin, 1992; Jacobs & Skocpol, 2010; Murray, 2007). In the last quarter of the century, tensions heightened in both the Reagan and the Clinton administrations. When President Obama took office in 2008 pledging to reform healthcare, partisan polarization took off. Reform became a polemical dogwhistle and a battle to be waged in a counterfactual context. The complexity of the system facilitates a disavowal of the public ‘socializing’ transfers that underpin it. It is a system dominated by private employer-led, large group insurance but there is a vast range of state subsidies and incentives to employers as well as state funded and administered systems catering for the aged, veterans, children and the very poor.

Solidaristic elements persist even within a predominantly privatized insurance system. Insurance is solidaristic because it transforms entities into grouped, risk-bearing categories (McFall & Moor, 2018; Meyers & Van Hoyweghen, 2018). Risk has to be grouped because it cannot be calculated at an individual level. Instead individual risk profiles are derived from membership of defined groups. Invoking Frank Knight’s (1921) classic distinction between risk, which is a calculable property, and uncertainty, which is not, Ewald explains that risk ‘only becomes something calculable when it is spread over a population. The work of the insurer is, precisely, to constitute that population by selecting and dividing risks’ (1991, p. 203).

This selection and division of risk groups configures rather than dismantles solidarity. A purely distributive, or purely solidaristic, form of insurance in which nothing is known about individuals and no individual risk assessments are carried out, Abraham (1985) noted, may be possible in theory but it is not seen in practice. Instead, assessment drives contemporary insurance practice and this ‘necessarily limits the amount of risk distribution achieved by an insurance arrangement, because it uses knowledge about risk expectancies to set different prices for members of different groups’ (Abraham, 1985, p. 405). This classification of individuals into pools, in theory, could mean pools get smaller and smaller. Ericson, Barry and Doyle (2000) made a strong case that this unpooling was precisely what the private insurance industry was doing by the end of the twentieth century. But whether the industry could ever function *as* insurance, and not some other kind of finance, conceptually, infrastructurally or practically, with truly individual or personal risk classification is a confounding puzzle. Abraham wrote that no risk classification system ‘can classify and price individual risks with anything near complete accuracy; the future is too uncertain for that’ (p. 405) but that was in 1985, long before the personalizing tendency in everyday data encounters had begun to manifest.

Currently, individual risk assessment is practiced alongside distribution in private insurance. This is true whether the insurance pool is run for the private benefit of its members, proprietors and investors, for public, societal benefit or, more likely, some combination. US health insurers, known as ‘payers’, may be publicly or privately owned or one of several variants including mutuals and co-ops. These ownership arrangements however are not the only thing that complicates the definition of a *privatized* actuarial system. There are also the state funding transfers to be accounted for. Almost counterintuitively, the US government pays considerably more per head toward healthcare than the UK government, despite its single payer, free at the point of use, National Health Service. The Commonwealth Fund's 2015 calculations placed public spending on healthcare in the United States the third highest at $4,197 per capita.^1^ The US system also ranks as one of the world’s most expensive ways of delivering indifferent health outcomes.

The idea of solidarity sets in motion the practical task of organizing the distribution of risk, but it does not supply the recipe. Rules surrounding membership, contribution, entitlement and reimbursement have to be made to fit distinctive political and cultural logics. Different national systems and the forms of public, redistributive transfer that underpin them, are shaped by a mess of political and historical contingencies. In the next section, I discuss how the Affordable Care Act promotes behavioural personalization while simultaneously placing regulatory barriers in the way of pricing personalization.

## The Affordable Care Act as redistributive regulation

**Figure 1:**
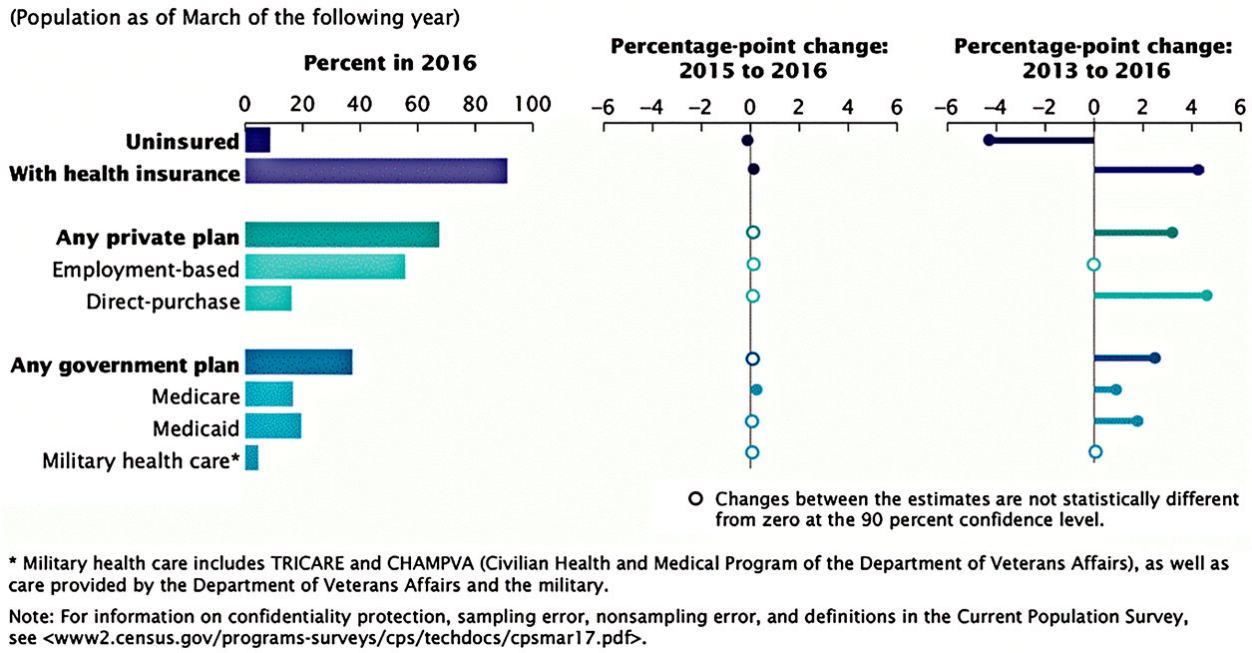
Percentage of people by type of health insurance coverage and change from 2013 to 2016 *Source*: US Census Bureau, 2017.

The United States has a byzantine healthcare system structured in four main parts (See Figure 1). Healthcare provision is generally, though not always, separated from payment. For the majority of Americans payment is made through private, employment-related insurance. This means that US healthcare is often categorized as a privatized insurance system. While this is true, it is also misleading. The label obfuscates the government’s funding role in two of the largest branches of US healthcare: Medicare and Medicaid. Medicare, the program for the over 65s; and Medicaid, the program for those on very low income or living with disability, are publicly funded and accounted for around 40 per cent of coverage in 2016.

That these government programmes occupy such a large share of a nominally private system is peculiarly in keeping with the politically fragile and fractious history of US healthcare. This history has been debated extensively (Dobbin, 1992; Jacobs & Skocpol, 2010; Murray, 2007) but the takeaway point is that US healthcare is not so much a system that has refused funding by social transfers as it is one that has produced extraordinarily complex means of operating and identifying them. Since the 1930s, US healthcare reform has been piecemeal and painful. The financing for the original 1935 Social Security blueprint was subject to almost continuous disruption until the 1950s and beyond, and the financing of Medicare enacted in the 1960s was not stabilized until the Reagan administration (Skocpol, 2010). Healthcare policy reform became effectively a proxy for the partisan idea of free individuals standing against an interventionist state. Reform becomes ‘political theater, cleverly scripted to provoke media coverage, rev up partisans, and convince uncertain or uninformed voters that something big and scary still remains at issue’ (Skocpol, 2010, p. 1289).

This atmosphere, combined with a poorly understood, highly complex and variable system, promotes a situation in which voters are apt to mobilize against their own interests. In the protests accompanying Obama’s reforms, the phrase ‘keep the government out of my Medicaid’ began to appear on placards. This ‘useful ignorance’ (McGoey, 2012) was exploited by the Republican Party (also known as the GOP, for Grand Old Party) opponents of the ACA. It enabled them to position ‘Obamacare’ as a scheme a scheme that was partly modelled on a Republican scheme (Mick Romney’s ‘Romneycare’). Labelling the ACA Obamacare transformed the legislation into a signal that the first black President meant to have government interfere in people’s most intimate rights to live – and die – as they chose. The most extreme manipulation of the fears this provoked was a Koch brothers funded commercial featuring a prone woman awaiting a clinical examination when approached by a speculum-bearing Uncle Sam.^2^ In this atmosphere, the years of partisan deadlock, and the federal shutdown that followed in 2014, were predictable. The vast governmental machinery required to administrate healthcare is, like all infrastructures, silent until it breaks (Bowker & Star, 1999). This allowed the blame for the well-known and well-documented problems with US healthcare to be volleyed around in a precursor of Trump’s post-factual politics.

Those who do not qualify for Medicare or Medicaid, and are not covered by their employers, can be accommodated by direct purchase in the ‘individual and small group market’. This is where the gaping hole in cover exists. The uninsured population varied between almost 40 per cent prior to the ACA, lowering to around 29 per cent, after the ACA’s full provisions came into force.^3^ The uninsured rate has begun to rise again with the Trump administration’s shambolic weakening of the Act’s provisions.^4^ Even among those with cover, underinsurance is widespread and medical expenses are the leading cause of personal bankruptcy. The failures in the individual/small group market on which this paper focuses, presented the major, though not the only challenge^5^, the ACA addressed. High proportions of people opted out of this market because of pricing and access barriers. Those with ‘pre-existing conditions’ were met with prohibitive prices even if they could find an insurer willing to offer cover. The ACA addressed this in three main ways. It imposed detailed requirements on insurers offering health plans, it introduced the ‘individual mandate’ requiring the purchase of health plans and it offered a range of public subsidies to make them more affordable.

As Tom Baker (2010) notes, this was part of a longer trend away from an ordinary market approach towards a ‘fair share’ approach supported by taxation and market regulation. ‘Fair share’, though, was far too close to an incursion on individual freedom for Republican appetites and the ACA encountered a relentless barrage of legal challenges. Ironically, the same concerns about state incursion and subsidy coming from within the GOP’s Freedom Caucus of ultra conservatives and libertarians hobbled the Trump administration’s first attempts to replace the ACA with the American Health Care Act. At the time of writing, an albeit weakened ACA remains in force and has accomplished some measure of redistribution. The administration’s many botched attempts to fulfil the ‘replace and repeal’ campaign promise has foundered repeatedly on – who knew? – the extraordinary complexity of interests and regulation involved in delivering healthcare policy. Trump has settled on significantly weakening the Act’s protections including repealing the individual mandate and permitting the sale of inexpensive ‘skinny’ plans with limited protections.^6^ What remains in place contains important lessons about the paradox inherent in personalizing solidarity.

Tackling the malfunctioning individual market meant finding a means of covering the 41 million people estimated to be uninsured at the end of 2013.^7^ The means selected were federal subsidies and the individual mandate that required all eligible Americans to have basic health coverage or risk a penalty known as the Shared Responsibility Payment (SRP). State marketplaces were set-up through which providers could offer ‘Qualified Health Plans’ (QHPs) featuring pre-defined essential health benefits, established limits on cost-sharing (that is, the amount individuals pay for directly through deductibles, co-payments and out-of-pocket expenses) and identical premiums irrespective of pre-existing conditions.^8^ QHPs come in different categories – bronze, silver, gold, platinum and catastrophic – and feature levels ranging from 60 to 90 per cent of cost-sharing with providers. Subsidies are available for those with incomes between 100 and 400 per cent of the Federal Poverty Line and, with limited exceptions for age and smoking, insurers cannot charge more for pre-existing conditions.

Prior to the ACA insurers could discriminate against those with pre-existing conditions. They charged higher premiums and refused cover to individuals whose health status meant they would require ongoing and expensive care. Pre-existing conditions and ‘lifetime limits’ on care costs have since become shorthand for the public debate surrounding the repeal efforts.^9^ In prohibiting pricing and access discrimination the ACA sought to collectivize entrenched individualistic ideas about fairness and responsibility. The link made between fairness and healthcare consumption is in line with the actuarial fairness approach outlined by Arrow (1963). Here fairness is the outcome of statistical risk pricing. The ACA alternative introduced a ‘behavioural’ approach (Meyers, 2018) including new responsibilities to pay a ‘fair share’ of the costs of the entire pool and be ‘as healthy as you can’. The responsibility to be healthy is promoted by the provision of access to preventative care and treatments for chronic, preventable disease. Smoking is not defined as a pre-existing condition and smokers face surcharges but they must also be offered free cessation therapy.^10^ This emphasis on behavioural responsibility is a great fit with data-driven healthcare innovations including wearable self-tracking devices and apps. These solutions are usually accessed outside traditional healthcare settings and are much more likely to be consumer retail purchases than prescriptions. That, as Neff and Nafus (2016) explain, places them outside of both the regulations that govern medical devices and the legal protections that apply to data gathered in healthcare settings.^11^

On the provider side, the ACA attempts to promote health and wellness by introducing Accountable Care Organizations (ACOs). ACOs feature a reimbursement model based on the *quality* of outcomes rather than the *quantity* of services. Fee-for-service remains the dominant payment model but in ACOs reimbursement is tied to quality metrics and reductions in the total cost of care via prevention. ACOs must ‘define processes to promote evidence-based medicine and patient engagement, monitor and evaluate quality and cost measures, meet patient-centeredness criteria and coordinate care across the care continuum’ (Gorrell, 2016, pp. 4–5). That reimbursement systems are drawn into relentless gaming where payer and provider interests collide in the classification, coding and reimbursement of care in a policy environment that is constantly adjusting incentives, has been well documented (Bowker & Star, 1999; Gerson & Star, 1991; Pasquale, 2013). For Neff and Nafus (2015), overt gaming has been ramped up in the financialized environment that health care reforms, including the ACA, operate within. The ‘medical loss ratio’, often used to express the difference between medical and administrative costs, has also been invoked as a signal to investors. A payer with a low medical loss ratio may be read as one with a healthier, and less costly, pool.

The ACA directly addresses this issue by requiring large health plans to maintain a medical loss ratio of 85% or higher. Plans with medical loss ratios below this threshold will be required to pay a rebate to the employers or individuals who purchased the plan. The purpose of the law is to ensure that health insurance premiums go to paying for medical services and to limit the proportion spent on profits and other administrative expenses. (Mulligan, 2015, p. 45)

As Mulligan explains, this produced an immediate burst of creativity as payers, once bent on restricting what counted as reimbursable medical care, began pushing for an enlarged definition. The resulting ‘payment policy arms race’ has payers and providers both using data coding specialists to either provide, or avoid, a rationale for claims denial.

This adds up to an environment in which datafied individualization of health responsibility appears like an inevitability. As Schüll notes, in the buzzing digital health scene, new technologies and the ACA have been presented as a ‘dynamic duo’ working together and ‘compelling insurers, health care providers and consumers to cut costs … shifting the management of chronic conditions like diabetes and heart disease away from hospitals and doctors and into the hands of patients themselves’ (2016, p. 318). Whatever benefits this technological and legislative communion in fostering patient-centred care may offer, there are intrinsic risks that data driven personalization could also mean targeting, discrimination and exclusion of minority and ‘underserved’ groups. Insurance companies thus might use the data ‘to manage our health through incentives, punishments or rewards that exert tight control over our daily activities’ (Neff & Nafus, 2016, p. 135). Lupton (2016a) cautions

Insurance and credit companies are scraping big data sets to develop customer profiles, with the result that disadvantaged groups suffer further disadvantage by being targeted for differential offers or excluded altogether because they are not viewed as profitable or as poor credit risks. Data brokers in the United States use available personal data to calculate certain predictive ‘health scores’ on patients with the help of digital data; such scores include the ACA *individual health risk score*, which is used for assessing the risk factor for an individual who requires healthcare. (p.120, emphasis added)

That insurers are using wearables and smartphones to financialize new ‘big mother’ forms of nudging surveillance, is a widely circulated narrative. In September 2018, when the US insurance company John Hancock announced it would stop underwriting traditional life insurance to sell only ‘interactive’ policies that track fitness and health data, the product release was announced by Reuters and provoked a volley of free publicity. *The New York Times*, *Forbes*, *CNBC*, the *BBC* and *The Conversation* were among many covering the story. It even earned an unusually well-circulated response to an insurance story from academic Twitter with Kate Crawford, founder of the AI Now Institute at New York University, earning 1,807 retweets, 2,416 likes and 63 replies^12^ for a tweet remarking on the ‘endless trapdoors ahead: data inaccuracies, intentional gaming, constant intimate surveillance 24/7’ (Crawford *et al*., 2015; Lupton, 2016a).

The stakes are high after all. These devices, as Nafus puts it, ‘could yet become the very worst modernity has to offer – social control masquerading as science’ (2016, p. xii). Data ethicists remark on the disparity between people’s expressed privacy concerns and their data practices (Moats & McFall, 2018). Faced by long and detailed terms and conditions and a barely comprehensible technical vocabulary it is not surprising that many ‘click and accept’ rather than analyze what kind of uses their personal data might be put to. Personal data gathered from apps, devices and websites has already been monetized by stealth because it is so easy to bury permissions in plain sight and so difficult to figure out what is, and what is not, a permissible use. The Google Deepmind/ NHS Royal Free health data controversy, as excavated by Powles and Hodson (2017), is a great example of this. It is also a sign of the importance of the regulatory environment and the need for expertise, effort and attention to the specificities of data practices. In the case of insurance pricing, this means studying carefully how the tensions between risk assessment and distribution are being addressed. The role of the Individual Risk Score in the ACA’s model is a good place to start.

### The individual risk score: a redistributive personalization

That insurers offering QHPs cannot deny coverage or charge premiums based on health status might imply that risk distribution presides over assessment in the ACA. Insurance however creates tendencies toward risk selection, adverse selection and moral hazard that have, in practice, to be managed through some form of risk assessment. How these tendencies are defined and managed has been extensively debated (c.f. Ericson *et al*., 2000; Leaver, 2015). Risk selection, in general, is observed where insurers market to low risks, for example by developing products with low premiums and high deductibles like the ‘skinny plans’ introduced by the current administration. These are unlikely to appeal to those with expensive health conditions. Risk selection can also be accomplished by marketing that targets the affluent, educated and healthy indirectly. Adverse selection occurs because those who know themselves at greatest risk are considered more likely to buy insurance. Moral hazard in health insurance comes into play where the insured overconsume services or even become wilfully negligent of their health with an insurance pay-out in prospect. In the absence of any risk assessment to regulate such tendencies, pricing uncertainty can deter providers from offering plans or result in volatile premium pricing. Adverse selection could lead to ‘death spirals’ in marketplaces disproportionately populated by those in need of care, leading to higher premiums. To address these kinds of distortions the ACA introduced three actuarial programmes – risk adjustment, reinsurance and risk corridors – designed to counter adverse and risk selection and to stabilize premiums. These are outlined in Table 1.

**Table 1:** Summary of Risk and Market Stabilization Program in the Affordable Care Act.

	**Risk Adjustment**	**Reinsurance**	**Risk Corridors**
***What*** **the programme does**	Redistributes funds from plans with lower-risk enrollees to plans with higher-risk enrollees	Provides payment to plans that enroll higher-cost individuals	Limits losses and gains beyond an allowable range
***Why*** **it was enacted**	Protects against adverse selection and risk selection in the individual and small group markets, inside and outside the exchanges by spreading financial risk across the markets	Protects against premium increases in the individual market by offsetting the expenses of high-cost individuals	Stabilizes premiums and protects against inaccurate premium setting during initial years of the reform
***Who*** **participates**	Non-grandfathered individual and small group market plans, both inside and outside of the exchanges	All health insurance issuers and self-insured plans contribute funds; individual market plans subject to new market rules (both inside and outside the exchange) are eligible for payment	Qualified Health Plans (QHPs), which are plans qualified to be offered on a health insurance marketplace (also called exchange)
***How*** **it works**	Plans’ average actuarial risk will be determined based on enrollees’ individual risk scores. Plans with lower actuarial risk will make payments to higher risk plans. Payments net to zero	If an enrollee’s costs exceed a certain threshold (called an attachment point), the plan is eligible for payment (up to the reinsurance cap). Payments net to zero	HHS collects funds from plans with lower than expected claims and makes payments to plans with higher than expected claims. Plans with actual claims less than 97% of target amounts pay into the programme and plans with greater than 103% of target amounts receive funds. Payments net to zero
***When*** **it goes into effect**	2014, onward (Permanent)	2014–2016 (Temporary − 3 years)	2014–2016 (Temporary - 3 years)

*Source: Redrawn from* Kaiser Family Foundation, 2016.

Risk adjustment addresses selection bias by calculating Individual Risk Scores to determine the overall actuarial risk of plans. It transfers funds from plans with lower risk profiles to those with higher risk profiles and is the only provision that was designed to be permanent. Reinsurance is a temporary programme to stabilize individual market premiums by reducing the incentives for insurers to charge higher premiums due to uncertainty about the health status of enrollees. It provides reinsurance payments when plan costs cross a threshold called an ‘attachment point’, while if reinsurance contributions fall short of the amount requested for payments, then that year’s reinsurance payments decrease proportionately. Risk corridors promote accurate premiums by discouraging insurers from setting high premiums to hedge against uncertainty about who they enrol and what they might cost. The corridors set a target of 80 per cent of premium dollars to be spent on health care and quality improvement, insurers with costs 3 per cent less than the target were to pay into the programme and the collected funds were to be used to reimburse plans with costs more than 3 per cent. Payments, in the original statute, were not required to net to zero, and any increase in costs or revenues was to be borne or absorbed by the federal government. This provision was weakened by Congress in 2015 and 2016 amidst arguments against federal bailouts for the insurance industry. In June 2018, two ACA insurers lost a case against the federal government claiming they were entitled to payment under the risk corridor programme.

The marketplaces, as it turned out, have not been overly profitable. The individual mandate was aggressively challenged. In the 2012 Supreme Court case the mandate was compared to the government forcing Americans to buy broccoli.^13^ As a result, the Shared Responsibility Payment (SHP), the penalty for not complying, was soft, ranging from only $95 in 2014 to $695 in 2016. The present US government is due to abandon it in 2019.^14^ The SHP was cheaper than the cost of even the cheapest plans and was not a sufficient incentive for the targeted ‘young invincibles’. Those enrolling between 2014 and 2016 were disproportionately older, sicker and costlier, and with the reduction in federal funding of the risk corridors, the state markets in 2016 saw rising premiums and the steady withdrawal of plans and insurers, particularly by smaller players and co-ops.^15^

The case highlights the tensions between assessment and distribution, individual and collective responsibility, in insurance solidarity. Risk corridors, reinsurance and risk adjustment measures are all designed to help the market function and conform to policy expectations. As Zuiderent-Jerak and Egmond remarked of a risk adjustment system in the Dutch market, the aim is to ‘ensure that solidarity among the insured would not be at odds with competition between insurers’ (2015, p. 48). Here too, risk adjustment is a device to reconcile marketized health care and redistributive solidarity. The Individual Risk Score is therefore in the equivocal position of being *both* an instance of data-driven personalization, as Lupton (2016a) suggests, *and* an element in how the ACA practices solidarity. In its aim to redistribute funds from plans with lower-risk to those with higher-risk enrollees, risk adjustment is a technique designed to stabilize premiums and widen access to health cover. Individual Risk Scores – drawn from age, sex and diagnoses – are de-identified and assigned to each enrollee to determine the average risk score in each QHP. This allows plans with lower actuarial risk to make payments to those with higher risk.

Once individual risk scores are calculated for all enrollees in the plan, these values are averaged across the plan to arrive at the plan’s ***average risk score.*** The average risk score, which is a weighted average of all enrollees’ individual risk scores, represents the plan’s predicted expenses. Under the HHS methodology, adjustments are made for a variety of factors, including actuarial value (i.e. the extent of patient cost-sharing in the plan), allowable rating variation, and geographic cost variation. Under risk adjustment, plans with a relatively low average risk score make payments into the system, while plans with relatively high average risk scores receive payments. (Kaiser Family Foundation, 2016)

That these devices struggled to stabilize insurance markets and accomplish the ACA’s redistributive goals is partly a function of how the law has fared in the bitter US policy environment. As I’ll argue next, the ACA may have created incentives for digital apps and wearables to nudge people towards health goals, but that does not lead inevitably to their incorporation in insurance risk assessment.

## Misfits? Oscar Health, wearables and the infrastructural challenges of insurance pricing

In April 2015, *The New York Times* reported that Oscar Health had joined the ranks of ‘unicorn start-ups’, with a valuation above $1 billion, just 16 months after going live (de la Merced, 2015). Oscar was then valued at $1.5 billion after raising $145 million to enable it to expand outside New York and New Jersey, where it had gathered 40,000 customers by spring 2015. In February 2016, *Forbes* recorded a $2.7 billion valuation when Oscar had enrolled a modest 135,000 customers (Bertoni, 2016; Levy, 2017). The against-the-grain valuation is notable because Oscar lost $120 million in 2015, then $200 million in 2016, and while the firm says it is ‘after one of the largest markets in the US, one that is worth 20% of GDP’, it is a tiny player in US health insurance.^16^

Start-ups are rare in health insurance, the market is dominated by giants like United Health, Anthem, Cigna, Aetna and Humana, which are nevertheless cautious enough about market conditions to be immersed in attempts at further consolidation. Oscar was founded in 2013 to offer plans on the new ACA markets. These markets are themselves a small portion of the overall health insurance market which is dominated by the employers’ market covering 150 million people. In contrast, just 7 million people were covered through the ACA markets in 2014 and 12.7 million in 2016. This might not make for headline grabbing valuations, but the company has generated disproportionate attention ever since its launch. One reason for this is that Oscar has been marked out as a bellwether for the mix of disruptive innovation and ‘dataveillance’ that may lay a path toward personalized insurance pricing.

Oscar has all the right hallmarks. It was founded by Joshua Kushner, Kevin Nazeemi and Mario Schlosser, Harvard Business School and Stanford trained entrepreneurs and data scientists. It aims to transform ‘user experience’ by developing a more human and transparent interface with customers in a field notorious for complex, impenetrable pricing and billing practices (Schleifer, 2016). It offers free primary-care ‘tele-visits’, downloadable electronic health records, a navigable enrolment process with transparent costs and benefits and incentivized preventative care – and all visibly integrated in ‘full stack’ Silicon Alley product branding. Among these features, one stands out.

**Figure 2:**
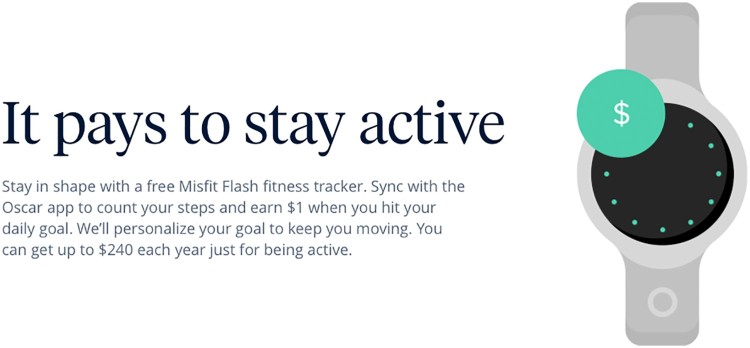
Oscar Health Misfit Scheme *Source*: Oscar Health screenshots, 17 April 2016.

In August 2014, the company announced it would begin offering members a free *Misfit* fitness wearable plus Amazon gift-card rewards for those who met individualized, algorithmically determined step-targets. This initiative drove much of the firm’s initial press coverage which drew parallels with in-car telematics devices to frame questions about the future prospects of price personalization.^17^ ‘What if’, Steven Bertoni (2014) asked, ‘thanks to wearables, health insurance began to work like car insurance where every health infraction (say a bar bender, Thanksgiving feast or sedentary Sunday of Netflix binge) hurt your health score and rocketed your health premiums?’ Schlosser countered that discriminatory pricing is illegal under the ACA and Oscar’s investment was calculated for risk reduction, not assessment: ‘if we can really get people to walk more, it will almost for sure have a huge impact on population health, and eventually health care costs, and that's certainly worth investing in’ (Fischer, 2014), see Figure 2.

Headlines continued throughout 2015 but the emphasis began to shift onto the risks the company faced as it expanded to Southern California and Texas for 2016 open enrolment.^18^ In 2016, the story was initially the disconnect between the valuation and the losses recorded in regulatory filings. By the summer a stream of articles investigated the ‘struggles’ and ‘headaches’ Oscar was encountering even before it withdrew from markets in Dallas and New Jersey in August.^19^ In stark contrast to the concern that wearable linked insurance would provoke an era of behaviour-based pricing, Oscar lost money, largely because its members were sicker than they expected. Enrolling sicker customers than were in the pre-ACA individual markets, according to a Blue Cross Blue Shield survey, was a feature across the ACA markets.^20^ Like most others, Oscar had priced aggressively low:

In an effort to attract customers, insurers put prices on their plans that have turned out to be too low to make a profit. The companies also assumed they could offer the same sort of plans as they do through employer-based coverage, including broad networks of doctors and hospitals. But the market has turned out to be smaller than they hoped, with 12 million signed up for coverage in 2016. Fewer employers have dropped health insurance than expected, for example, keeping many healthy adults out of the individual market. And among the remaining population, the insurers cannot pick and choose their customers. The law forces them to insure people with pre-existing conditions, no matter how expensive those conditions may be. (Abelson, 2016)

Oscar’s response to these challenges suggests that wearable data is not seen as a short route to price personalization. As Schlosser maintains, the regulations surrounding pre-existing conditions in the ACA prevent price personalization. There are other insurance markets with fewer regulatory barriers and Oscar has made it clear that the ACA markets are an entry point not an end goal for the firm. So, in theory, the Misfit scheme could function as a data learning exercise for the firm, enabling it to model relationships between wearable data and health care costs and use this to inform risk assessment in other markets.

This seems plausible but there are significant problems with it. Health risk assessment is much more complicated than driving risk assessment. In-car telematics provide constant and comprehensive data-sets that demonstrate strong correlations between accidents and a limited set of variables in individual driving behaviour, for example, number of hours driven, time of journey, speed, late braking, etc. Wearables and apps provide partial data that approximate movement, and often heart rate, from accelerometers and other sensors. The data are partial, because users don’t always carry their devices, falsifiable because trackers don’t know who or what is carrying the device, and of limited risk assessment value because there is no clinically established relationship between movement data and health. There is data, much of it gathered by Discovery Ltd., the parent company of the market leading Vitality franchise, including a Harvard Business School Case (Porter *et al.,*^2014^), that supports a relationship between activity tracking and health outcomes but this, like risk more generally, is meaningful at the level of populations but not really at that of individuals.

This being the case, why are health insurers interested in behavioural tracking and incentive schemes? One likely reason is that these schemes work as an extended and targeted form of promotion. This is borne out by the distinctiveness of Oscar’s brand. Writing in *Wired*, Steven Levy (2017) noted that everything from Oscar’s url (‘hioscar.com’) to its Apple-esque packaging ‘sends out a millennial dog whistle: *Come to me, my mobile-first darlings’*. The free Misfit tracker and the Amazon gift-cards target younger, hipper buyers – a group with a not-coincidentally lower probability of ill-health. Personalization, as Moor and Lury (2018) argue, is never just personal, it involves generalization and the production of ‘types of Persons’. The tracker and gift-cards are co-varying elements of the marketing mix, ‘since it is hard to separate the sense in which the benefits they offer are a form of promotion from the sense in which they are a form of pricing’ (2018, p.507).

Oscar’s claim to have ‘run the numbers’ suggesting returns in the form of lower health costs from the Misfit scheme rings hollow. A more likely calculation was that the device would do no harm and might do some small good to members’ health. The real benefits for Oscar would accrue if the scheme attracted younger, fitter customers who are, as Neff and Nafus (2016) note, the main users of wearables. This does not mean the scheme has no connection with pricing. Wearable programmes have risk selection-like characteristics (Arentz & Rehm, 2016) and this does impact on pricing but not by personalizing it. Making profits in health insurance means pricing to cover the projected costs of healthcare services consumed, at a level that attracts sufficient numbers of the right sort of customers and is compliant with policy regulation. That is a much tougher order than combining wearables and slick branding. To create commercial value in insurance requires what Van Hoyweghen calls an ‘intermingling of economic, managerial, accountancy, actuarial and medical knowledges, figures and tools’ (2014, p. 347). Oscar’s inflated valuation is what enables it to sustain its losses and although the level is unusual, insurance company margins are always partly a matter of how they are capitalized. Profit is derived at least as much from understanding the ‘time value of money’ as accurate risk pricing (Ericson *et al*., 2000; McFall, 2014).

Oscar’s response to its 2016 losses also points to how marginal self-tracking data is to cost control. It began by raising premiums by an average of 18.6 per cent^21^, reflecting the industry experience that ACA markets were under-priced. At the same time, it modified the product itself. The received wisdom in employer markets is that patient choice is sovereign and insurers in the ACA’s individual markets had largely followed the same principle. In 2016 it became clear that the companies which fared best were those with strong cost control, lower cost providers and limited networks. Oscar’s 2017 strategy cut back drastically on the number of care providers it contracts with to gain more control over pricing and patient experience. Their new, narrow network approach includes contracts with a limited, but prestigious, set of providers. They opened a clinic with Mount Sinai Health System featuring a yoga studio, wellness centre and receptionists/greeters trained to foment collaborative spirit by standing alongside people as they log their arrivals on iPads. Like the Apple stores it references, the clinic is the most visible face of Oscar’s ‘integrated system with a seamless flow of data and collaboration from the concierge teams to general practitioner to specialists to hospitals’ (Levy, 2017).

**Figure 3:**
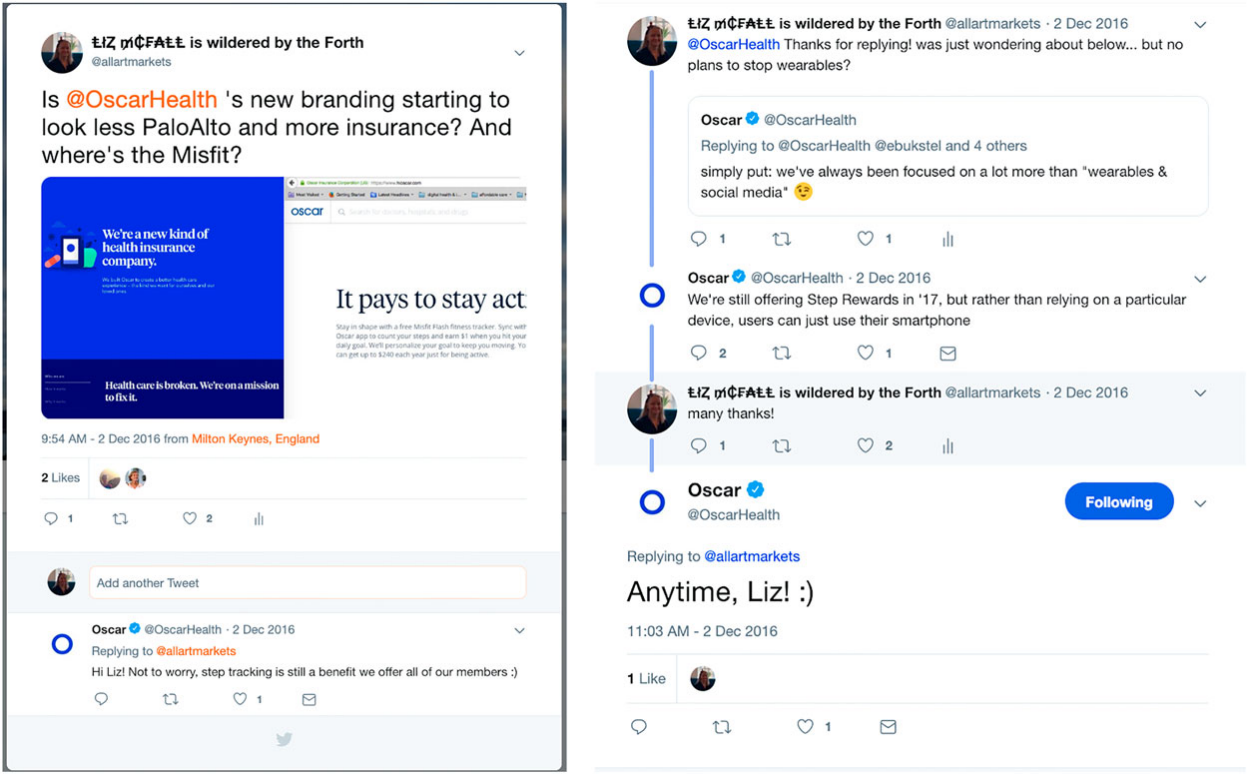
Oscar Health Twitter exchange, December 2016

This data must also flow back through billing, claims and enrolment processes to allow Oscar to work on the complex algebra that links the population appealed to, the population insured, the health costs incurred, claimed and reimbursed, the prices charged, the federal subsidies issued, then all the way back again. Big data integration, analyses and exchange, including the analysis of patient data, are at the centre of Oscar’s strategy but there is very little to suggest that wearable data is part of this mix. In a final mark of their limited role at Oscar, while the firm continues to offer fitness incentives, it no longer offers the *Misfit* and has, since 2016, steadily dropped its public association with ‘wearables and social media’, see Figure 3.

This move took place in an environment of uncertainty which reached its peak when Trump was elected in November 2016. A week after the election Oscar posted ‘Our post-election thoughts on healthcare’.^22^ The post was designed to ease concerns that the Republican commitment to repeal the ACA would damage the company. In it, the company founders acknowledged that ‘the ACA catalyzed a market that is responsive to this sort of disruption’ but insisted that their plans had always been ‘to sell quality health insurance across any product line’. This may be so, but the election result likely hastened the company’s pivot towards the group market. As things have turned out the Trump administration’s disavowal of expertise has made repeal tougher to enact than it is to chant about. Oscar doubled its presence in ACA markets offering plans in nine states for 2019 open enrolment and this, not group insurance, remains the core of their business. An interesting position for a company which is delicately snared in this policy context. Joshua Kushner is the brother of Jared, senior adviser and son-in-law to President Trump.

## Concluding comments

ACA markets highlight the stubbornness of the infrastructures of solidarity in healthcare payment. Self-tracking wearables have been cast as disruptive objects implicated in a divisive and discriminatory personalization of healthcare. They seem emblematic of broader trends in the digitalization of healthcare that for some, for example in quantified self and patient led care communities, present opportunities for empowerment and democratization (Neff & Nafus, 2016; Ruckenstein & Pantzar, 2015). For others, self-tracking practices are a characteristic of an emergent form of biopolitical governance, part of both a neoliberal redistribution of responsibility from the state to the individual and the extraction of monetary value from human life (Lupton, 2016a, 2016b). This argument, plausible on the surface, is much less so when self-tracking devices and practices are located within the conceptual, regulatory and infrastructural frameworks underpinning US health insurance.

Conceptually, insurance organizes solidarity across groups by balancing risk distribution and assessment. Personalizing solidarity to make risk an assessed and priced property of individuals would tip the balance so far towards assessment that it is not clear that any eventual product would qualify *as* insurance. Baseline definitions of insurance vary but almost all include the spread, distribution or transfer of risk, as a priceable entity, between insureds and insurers. A contract that uses self-tracking data to price an individual’s access to healthcare employs neither risk nor distribution as defined in insurance practices. Wearable surveillance that has financial and access consequences is a provocative future scenario but not an especially likely one for insurers to pursue seriously.

In regulatory terms, the existing ACA framework supports the adoption of data-driven behavioural technologies, it does not incentivise their incorporation in pricing or access decisions. Protections for pre-existing conditions, and the actuarial devices for reinsurance, risk assessment and risk corridors, purposefully prevent the use of any individual level data derived for pricing. Even as the Trump administration works on stealthily rolling back the hugely popular pre-existing condition protections,^23^ it remains unlikely that self-tracking data could be used to assess or price risk at an individual level for operational reasons.

Self-tracking data is used, alongside other health and wellbeing indicators like gym membership and health assessments, to promote insurance. Oscar Health is one example, but on a much larger international scale, the South African group Discovery Ltd. has been offering behaviour-based insurance products since the early 1990s. In the last decade Discovery have become the market leaders through the Vitality brand offered by a network of partners and franchisees. In their 2017 Annual Report, Discovery assert that data about customer engagement with its behavioural programmes in driving, health and life insurance, feeds into ‘dynamic risk pricing’. Dynamic risk pricing and price optimization are the industry terms that most closely resemble the concept of personalized pricing but there are instructive differences between them. Price optimization involves using ‘non-actuarial’ factors, including customer sentiment and propensity to buy. It is a strategy derived from the importance of online searches and comparison websites in insurance purchasing that produce harvestable data for customer analysis and modelling. This allows insurers to offer customers differential, dynamic prices according to their online characteristics and behaviour (Minty, 2016). This is a data-driven innovation in insurance pricing that is in widespread use but has had far less attention in critical scholarship than self-tracking data. It has not, however, escaped regulatory attention (Minty, 2017).

Underneath the conceptual, regulatory and infrastructural characteristics of insurance are challenges and opportunities that are not getting the attention they deserve. There are real obstacles to transforming self-tracking data into personalized risk categories but that does not mean there is nothing to see. Datafication processes, using data from hospital visits to web searches, are underway in insurance. These processes are black-boxed, lack transparency and sufficient regulatory oversight (Pasquale, 2013; 2015; Powles & Hodson, 2017). Yet, there is, as Kitchin (2016) points out, nothing inevitable about the current state of data regulation. Silicon Valley attitudes to the repurposing of data are at odds with the regulatory environment as expressed most recently in the European General Data Protection Regulation of 2018. GDPR regulation echoes earlier versions of ‘fair information principles’ particularly that of data minimization which states that data can only be used for the purposes for which it was collected. Closer attention to how this is interpreted in insurance practice would be a good target for critical data scholarship. Moving from group risk pricing to personalize risk pricing is a fundamental and problematic change for all parties to the insurance contract. It is far from inevitable in a slow-moving, reputation and price sensitive industry. Health insurance works in market spaces that are created by regulation that also protects characteristics - race, gender, genomic data and pre-existing conditions - from discrimination. As new possibilities for discrimination based on lifestyle tracking arise, so do new possibilities for regulation. Solidarity is an organized practice, it can be reorganized, maybe even personalized, but it has always to be regulated.

## Notes

1 http://www.commonwealthfund.org/publications/issue-briefs/2015/oct/us-health-care-from-a-global-perspective

2 See https://www.theatlantic.com/politics/archive/2013/09/creepy-anti-obamacare-ads-suggest-where-uncle-sam-wants-to-stick-it/279825/ - the commercial itself can be seen here https://www.youtube.com/watch?v=7KHjg6mtewI

3 48.6 million were uninsured in 2010 falling to 29.3 million in 2016 https://aspe.hhs.gov/basic-report/overview-uninsured-united-states-summary-2011-current-population-surveyhttps://www.kff.org/uninsured/fact-sheet/key-facts-about-the-uninsured-population/

4 As of September 2017, the Trump administration's numerous efforts to ‘repeal and replace’ the ACA continue. http://www.healthreformtracker.org/ahca-timeline/; http://khn.org/news/obamacares-history-littered-with-near-death-experiences/http://www.kff.org/interactive/proposals-to-replace-the-affordable-care-act/

5 There was also the contentious reform and expansion of Medicaid (Hertel-Fernandez *et al*., 2016).

6 See https://khn.org/news/marketplace-confusion-opens-door-to-questions-about-skinny-planshttps://www.kff.org/report-section/understanding-short-term-limited-duration-health-insurance-issue-brief/

7 http://kff.org/report-section/the-uninsured-a-primer-what-was-happening-to-insurance-coverage-leading-up-to-the-aca/

8 https://www.healthcare.gov/glossary/qualified-health-plan/https://www.healthcare.gov/coverage/what-marketplace-plans-cover/

9 One example was the ‘Jimmy Kimmel’ test. Senator Cassidy, of the Graham-Cassidy repeal bill of 2017, said that he would only sponsor legislation that would pass what he called the Jimmy Kimmel test. Kimmel is a US comedian whose son was born with a congenital heart defect requiring treatment that would have exceeded lifetime limits on costs in the first few months of his life. When the Graham-Cassidy bill came out, Kimmel agreed that the bill would pass a Kimmel test but a different one, one which meant that a child would get the care s/he needed – but only if its father was Jimmy Kimmel.

10 See https://www.healthcare.gov/preventive-care-adults/https://www.healthcare.gov/coverage/what-marketplace-plans-cover/http://obamacarefacts.com/obamacare-smokers/https://www.healthcare.gov/coverage/pre-existing-conditions/

11 Health Insurance Portability and Accountability Act 1996 https://www.hhs.gov/hipaa/for-individuals/guidance-materials-for-consumers/index.html .

12 Original tweet accessible at https://twitter.com/katecrawford/status/1042558396497649664

13 http://www.nytimes.com/2012/06/14/business/how-broccoli-became-a-symbol-in-the-health-care-debate.html?pagewanted=all

14 http://khn.org/news/faq-on-individual-insurance-mandate-aca/http://www.fool.com/investing/2016/08/14/4-reasons-your-obamacare-healthcare-premium-is-pro.aspx

15 Motley Fool reports only 12.6 per cent was paid out by federal government of the $2.87 billion requested to cover insurers’ losses on the marketplaces http://www.fool.com/investing/2016/08/14/4-reasons-your-obamacare-healthcare-premium-is-pro.aspx.

16 See https://www.crunchbase.com/organization/oscar#/entity

17 Google search results produced 237 stories about Oscar Health between April 2014-2016. See for example https://www.wired.com/2014/12/oscar-misfit/https://gigaom.com/2014/12/08/insurance-provider-oscar-will-reward-you-if-you-hit-your-step-goal/http://www.bizjournals.com/newyork/blog/techflash/2014/12/oscar-passing-out-fitness-trackers-incentives-to.htmlhttp://fortune.com/2014/12/09/oscar-health-insurance/http://gizmodo.com/an-insurance-company-will-pay-you-to-use-your-fitness-t-1668967153

18 https://www.bloomberg.com/news/articles/2015-09-03/for-health-insurance-startup-oscar-cute-ads-only-go-so-far; Lee (2015).

19 https://www.fastcompany.com/3055700/warning-trying-to-disrupt-health-insurance-may-cause-headacheshttp://www.vanityfair.com/news/2016/08/josh-kushner-oscar-health-obamacarehttps://www.bloomberg.com/news/articles/2016-08-23/insurance-startup-oscar-quits-markets-rethinks-obamacare-plans

20 https://www.bcbs.com/the-health-of-america/reports/the-evolving-affordable-care-act-marketplaces-the-2015-2016.

21 http://www.crainsnewyork.com/article/20160518/HEALTH_CARE/160519847/health-insurers-are-requesting-double-digit-bumps-for-obamacare-premiums-across-the-state-with-unitedhealth-seeking-a-45-6-hike

22 https://www.hioscar.com/news/our-post-election-thoughts-on-healthcare

23 https://www.kff.org/health-reform/issue-brief/ask-kff-karen-pollitz-answers-3-questions-on-trump-administrations-new-aca-waiver-guidelines/

## Notes

**Liz McFall** is Director of GovTech and Chancellor’s Fellow based in the Edinburgh Futures Institute and Sociology at the University of Edinburgh. She co-edited *Markets and the arts of attachment* with Franck Cochoy and Joe Deville (Routledge, 2017), is author of *Devising consumption: Cultural economies of insurance, credit and spending* (Routledge, 2014) and *Advertising: A cultural economy* (Sage, 2004). She is Co-Editor-in-Chief of the *Journal of Cultural Economy*.
